# Editorial: Diabetic neuropathy and its complications

**DOI:** 10.3389/fnins.2023.1267417

**Published:** 2023-08-14

**Authors:** Hung-Chen Wang, Cheng-Hsien Lu

**Affiliations:** ^1^Department of Neurosurgery, Kaohsiung Chang Gung Memorial Hospital, Chang Gung University College of Medicine, Kaohsiung, Taiwan; ^2^Department of Neurology, Kaohsiung Chang Gung Memorial Hospital, Chang Gung University College of Medicine, Kaohsiung, Taiwan

**Keywords:** diabetic neuropathy, stress hyperglycemia ratio, nuclei iron accumulation, quantitative thermal testing, axonal transport deficits

Many diabetic people experience diabetic neuropathy (DN), a frequent and crippling consequence of diabetes. In addition to hyperglycemia, the pathogenesis of DN includes intricate and multiple mechanisms that involve critical intracellular pathways, pro-inflammatory cytokines, neurons and satellite glial cells (Bishnoi et al., 2011; Jia et al., 2017; Cheng et al., 2021; Chen et al., 2022). This editorial synthesizes insights from four pioneering studies in 2023 that shed new light on the understanding and management of DN.

Feng et al. conducted a study to investigate the relationship between stress hyperglycemia ratio (SHR), an indicator of stress hyperglycemia, and inflammation biomarkers such as neutrophil counts and neutrophil-to-lymphocyte ratio (NLR) in acute ischemic stroke (AIS) patients. They discovered a definite correlation between neutrophil counts and NLR and SHR levels using data from 487 AIS patients. Higher neutrophil counts and NLR were found to be independent risk factors for increased SHR in patients with large-artery atherosclerosis and cardioembolism by subgroup analysis. The levels of these biomarkers did not significantly differ between patients with and without small-vessel blockage. According to the study's findings, neutrophil counts and NLR are positively correlated with SHR levels in AIS patients, and their linkage with SHR levels varied depending on the functional severity of the stroke and the etiology of the stroke.

In another study, Hu et al. investigated how type 2 diabetes mellitus (T2DM) patients' deep gray nuclei iron accumulation affects cognitive deterioration. The study evaluated the magnetic susceptibility values (MSV) in 29 T2DM patients and 24 age- and gender-matched healthy controls using a strategically recorded gradient echo sequence. The findings showed that all gray matter nuclei in T2DM patients had an increase in MSV of 5.1–14.8% and a significant decrease in whole-structural volumes of the majority of gray nuclei. Additionally, a strong association between MSV in the right globus pallidus and the results of the List A Long-delay Free Recall, indicating a deterioration in cognitive function, was discovered. The study came to the conclusion that cognitive impairment may be associated with excessive and heterogeneous iron accumulation in deep gray nuclei in T2DM patients.

In patients with prediabetes and type 2 diabetes, Huang et al. assessed the usefulness of quantitative thermal testing (QTT) as a technique for initial screening and continuous assessment of diabetic sensorimotor polyneuropathy (DSPN). They first identified 89 patients (22%) with DSPN using the Toronto Clinical Neuropathy Score (TCNS) and electrophysiological testing, of which 29 regressed to no DSPN and 20 advanced to DSPN over a year. They discovered a substantial correlation between TCNS and nerve conduction scores as well as warm and cold detection thresholds in the hands and feet. The presence of DSPN was independently correlated with the foot Cold Detection Threshold (CDT). The four different DSPN statuses were statistically significant for each metric across time. Finally, DSPN-related nerve damage over a year can be progressive or reversible and can be objectively assessed by nerve conduction investigations and QTT. The foot CDT may be used as an early screening tool for DSPN.

Lastly, Yang et al. present a review focused on the role of axonal transport deficits in the progression of diabetic peripheral neuropathy (DPN). DPN is a prevalent metabolic disorder in hyperglycemic patients that puts a heavy financial burden on healthcare systems and has the potential to cause amputations and neuropathic pain. The majority of current treatments focus on symptoms rather than fundamental reasons. Patients with long-term diabetes mellitus (DM) frequently display axonal transport impairment, which is thought to contribute to or aggravate DPN. The review investigates the processes of nerve fiber loss, decreased nerve conduction velocity, and delayed nerve regeneration in relation to axonal transport impairment and cytoskeletal alterations brought on by DM. The authors emphasize the critical need of prompt and effective correction of axonal transport deficits for managing peripheral neuropathies, arguing that knowing these pathways is key for preventing DPN worsening and generating new treatments.

In summary, these studies highlight the complexity of DN and each one adds to our understanding of its pathophysiology and management. Despite these developments, further study is still required to properly understand these complex connections and turn them into more effective DN therapies.

## Author contributions

H-CW: Writing—original draft, Writing—review and editing. C-HL: Conceptualization, Writing—review and editing.
